# Natural Occurrence of Main Mycotoxins in Herbs and Spices Commercialized in Italy

**DOI:** 10.3390/foods14111889

**Published:** 2025-05-26

**Authors:** Katia Gialluisi, Nada El Darra, Maria Giovanna Nicoletti, Michele Solfrizzo, Lucia Gambacorta

**Affiliations:** 1 Institute of Sciences of Food Production (ISPA), National Research Council of Italy (CNR), Via Amendola 122/O, 70126 Bari, Italy; katia.gialluisi@cnr.it (K.G.); michele.solfrizzo@ispa.cnr.it (M.S.); 2 Department of Nutrition and Dietetics, Faculty of Health Sciences, Beirut Arab University, Tarik El Jedidah—Beirut, P.O. Box 115020, Riad El Solh, Beirut 1107 2809, Lebanon; n.aldarra@bau.edu.lb; 3 Ladisa Srl-Ristorazione, Via Lindemann Z.I, 70132 Bari, Italy; mariagiovannanicoletti@pec.it

**Keywords:** occurrence, spices, herbs, multi-mycotoxins, Italy, UPLC-MS/MS

## Abstract

A total of 98 samples of spices, herbs, and mixtures commercialized in Italy were analyzed for 11 mycotoxins, regulated and non-regulated. The occurrence of 1–4 mycotoxins was found in 84% and 60% of spice samples and herb samples, respectively. Spices were the most contaminated matrix. Total aflatoxins were detected in 5% and 9% of herbs and spices, respectively, and ochratoxin A was detected in 14% of spices and not at all in herbs. Only one sample of spices (cloves) showed an AFB_1_ value (9.0 µg/kg) higher than the limit. No sample of herbs and spices had an OTA content beyond the limit. Within the non-regulated mycotoxins, ZEA was the most commonly occurring, and its mean levels in positive samples of spices ranged from 4.6 µg/kg (ZEA) to 1813.9 µg/kg (FB_1_). The mean levels of FB_2_ and ZEA in positive samples of herbs were 131.4 µg/kg and 2.5 µg/kg, respectively. The mean levels of non-regulated mycotoxins in positive samples of herbs-and-spices mixtures ranged from 2.6 µg/kg (ZEA) to 1071.7 µg/kg (FB_1_). The most contaminated herbs and spices were garlic and basil, respectively. This study provides a significant amount of information on the natural occurrence of multi-mycotoxins in herbs and spices consumed in Italy, which will be useful for the future regulation of some mycotoxins, in particular, FBs.

## 1. Introduction

Mycotoxins are toxic secondary metabolites produced by different species of toxigenic filamentous fungi that belong to the *Aspergillus*, *Penicillium*, and *Fusarium* genera [1]. Nearly 400 types of mycotoxins have been discovered and are generally categorized into groups based on structural similarities and their major toxic effect [2]. The literature indicated that aflatoxins (AFs), ochratoxin A (OTA), fumonisins (FBs), deoxynivalenol (DON), T-2 toxin (T-2), HT-2 toxin (HT-2), and zearalenone (ZEA) can contaminate herbs and spices [3,4]. High-humidity and -temperature conditions during pre-harvest and post-harvest create a conducive environment for fungal proliferation and subsequent mycotoxin biosynthesis. Inadequate storage conditions, such as prolonged retention periods and ineffective drying processes, markedly enhance the likelihood of mycotoxin contamination [5]. Geographical origin significantly influences mycotoxin prevalence in herbs and spices, as regional environmental and agricultural conditions contribute to varying contamination levels [6]. The EU established maximum tolerable limits only for aflatoxins and ochratoxin A in certain spices. These regulations are based on toxicological data, survey data, and analytical methodologies [4]. AFs are the most toxic group of mycotoxins that are produced by some *Aspergillus* species (*A. flavus* and *A. parasiticus*) [7]. Among 18 different types of aflatoxin, aflatoxin B_1_ (AFB_1_) is the most extremely toxic, mutagenic, and carcinogenic type [8,9]. AFB_1_ and naturally occurring mixtures of AFs were classified by the International Agency for Research on Cancer (IARC) (1993) as group 1 carcinogens (carcinogenic to humans) [10]. OTA is produced by *P. verrucosum*, *A. niger*, *A. ochraceus*, and *A. carbonarius* [11] and can contaminate all types of cereals and derivates but also coffee, cocoa, grapes, soy, legumes, beer, and spices [12,13,14] prior to harvest or, much more commonly, during storage [15]. The IARC classified OTA as a probable human carcinogen (group 2B) based on sufficient evidence for carcinogenicity in animal studies and inadequate evidence in humans (IARC, 1993) [10]. The EU established maximum levels for AFs in foodstuffs according to Commission Regulation (EC) No. 1881/2006 [16] amended by Regulation (EU) No. 165/2010 [17], which set a legal limit of 5 µg/kg for AFB_1_ and 10 µg/kg for the sum of aflatoxin B_1_ (AFB_1_), aflatoxin B_2_ (AFB_2_), aflatoxin G_1_ (AFG_1_), and aflatoxin G_2_ (AFG_2_) in spices, including *Capsicum* spp. (dried fruits thereof, whole or ground, including chilies, chili powder, cayenne, and paprika), *Piper* spp. (fruits thereof, including white and black pepper), nutmeg, ginger, turmeric, and mixtures of species containing one or more of the mentioned spices [17]. Regarding OTA, Commission Regulation No. 1881/ 2006 [16] was recently amended by Regulation (EU) No. 1370/2022 to revise OTA levels in foodstuffs [18]. The EU Commission 2022 established a limit of 15 µg/kg for spices, including dried spices, ginger, and mixtures of spices. A limit of 20 µg/kg was established for the *Capsicum* spp. (dried fruits thereof, whole or ground, including chilies, chili powder, cayenne, or paprika) and licorice root, including as an ingredient in herbal infusions. Furthermore, a limit of 10 µg/kg has been indicated for dried herbs [18].

Currently, the European Union has not set maximum permissible levels for major mycotoxins such as fumonisins (FBs), deoxynivalenol (DON), and zearalenone (ZEA) in spices, although these mycotoxins are regulated in other food commodities under Commission Regulation (EC) No. 1881/2006 and subsequent amendments, based on the EFSA risk assessments. Several studies utilized liquid chromatography coupled with tandem mass spectrometry (LC-MS/MS) to enable the simultaneous detection of multiple mycotoxins in a single analytical run [19,20,21]. However, no comprehensive studies have been conducted on the occurrence of multi-mycotoxins in different types of herbs and spices commonly consumed in Italy. Two studies focused exclusively on regulated mycotoxins in spices, aromatic herbs, herbal teas, and medicinal plants marketed in Italy. Additionally, only one study has examined multi-mycotoxin contamination in black pepper produced in Italy, while a related investigation involving multi-mycotoxin determinations was conducted on herbs and spices consumed in Lebanon [4,19,22,23]. This study aims to evaluate the occurrence and levels of both regulated and non-regulated mycotoxins in a range of spice and herb samples commercialized in Italy. These results serve as a preliminary but important basis for assessing human dietary exposure to multiple mycotoxins from these products. Furthermore, the findings highlight the necessity for additional studies to establish safe limits for currently non-regulated mycotoxins, thereby informing future risk assessments and regulatory actions to protect human health.

## 2. Materials and Methods

### 2.1. Sampling

Ninety-eight different kinds of spices, herbs, and mixtures of herbs and spices were randomly collected in 2018 in different types of stores of Bari and neighboring countries in Italy, i.e., supermarkets, discount stores, and traditional shops. Of these, 65 samples were of single spices and 5 samples were spice mixtures, 19 samples were of single herbs, and only 1 sample was an herb mixture. At last, there were 8 samples of herbs-and-spices mixtures (Table 1). All the samples were brought to CNR-ISPA, where they were finely ground, mixed, and prepared for the multi-mycotoxin extraction.

At IRSA-CNR of Bari, the samples were analyzed by UPLC-MS/MS for AFB_1_, AFB_2_, AFG_1_, AFG_2_, OTA, FB_1_, FB_2_, T-2, HT-2, ZEA, and DON.

### 2.2. Chemicals and Reagents

For the calibration curve, commercially available standard solutions were used. The standard solutions were purchased from Romer Labs Diagnostic (Tulln, Austria). For fumonisins, separated standard solutions of FB_1_ (50 μg/mL) and FB_2_ (50 μg/mL) in acetonitrile/water (50:50, *v*/*v*) were used. The other standard solutions OTA (7.3 μg/mL), HT-2 (100.2 μg/mL), AFB_1_ (2.0 μg/mL), AFB_2_ (0.5 μg/mL), AFG_1_ (2.01 μg/mL), AFG_2_ (0.5 μg/mL), T-2 (100.2 μg/mL), ZEA (100.2 μg/mL), DON (100.3 μg/mL) were prepared in acetonitrile (ACN). Chromatography-grade methanol (MeOH), ACN, glacial acetic acid, and ammonium acetate were sourced from Sigma-Aldrich (Milan, Italy). Ultrapure water was generated using a Milli-Q system (Millipore, Bedford, MA, USA).

### 2.3. Determination of Mycotoxins

#### 2.3.1. Mycotoxins Extraction

The UPLC-MS/MS method previously described [4] for multi-mycotoxin determination was used herein. Briefly, 5 g of ground spices, herbs, or mixtures weres first extracted with ultrapure water, shaken for 60 min using an orbital shaker, and centrifuged for 10 min. Then, an aliquot of 17.5 mL of aqueous extract was filtered and collected (extract A). A volume of 17.5 mL of methanol was added to the remaining solid material and the samples were extracted again by shaking for 60 min and centrifuged for 10 min. The extract was filtered and collected (extract B). An equal volume (1 mL) of extract A and extract B were mixed and vortexed for 20 s. An overnight cold precipitation (4 °C) was performed for gentle cleaning of the sample. Then, the sample was diluted with adequate volumes of mobile phase solution and filtered through a 0.45 μm regenerated cellulose filter. A volume of 10 μL was analyzed by UPLC-MS/MS. For some of the more fiber-rich samples, a larger volume of water (up to 75 mL) was required for the first extraction due to their high water absorption capacity. In these instances, the volume of methanol used for the second extraction was increased proportionally (respecting the ratio of 70:30, *v*/*v*, MeOH:H_2_O). In addition, some samples were very oily, and only one extraction was carried out using 25 mL of a 70:30, *v*/*v*, MeOH:H_2_O mixture. Then, these samples were shaken for 60 min, centrifuged, refrigerated overnight, and lastly filtered and analyzed. A mixture of eight herbs and spices (cumin, cardamom, primula, basil, and a mix of four spices) was used to prepare the extracts for matrix-assisted calibration solutions. These five samples were mixed and ground, and aliquots of 5 g were used for matrix-assisted calibration solutions.

Standard solutions of OTA, FB_1_, FB_2_, HT-2, T-2, ZEA, DON, AFB_1_, AFB_2_, AFG_1_, and AFG_2_ were used to prepare seven mixed calibration solutions at varying concentrations, in dried extracts of herbs-and-spices mixtures samples. Standard dilutions were performed using ACN. The spiking levels, recoveries, and repeatability (RSDr) of the 11 mycotoxins were previously calculated by [4] and are reported in Table 2.

The method was applicable for the analysis of herbs and spices as recoveries ranged from 60% for AFB_1_ to 105% for FB_2_, and the mean repeatability (RSDr) of results ranged from 1% for FB_1_ and HT-2 to 23% for ZEA. The mycotoxin quantification was performed using matrix-assisted calibration curves. The calibration curves were constructed using dried sample extracts obtained from herbs-and-spices mixtures (as described above). The limits of detection (LOD) and quantification (LOQ) of each mycotoxin were calculated as a signal-to-noise ratio of three and six, respectively (Table 2). The noise was measured immediately before or after the peak of each analyte.

#### 2.3.2. LC-MS/MS Equipment and Calibration

UPLC-MS/MS analyses of multi-mycotoxins were performed by using a triple-quadrupole API 5000 system (Applied Biosystems, Foster City, CA, USA), equipped with an ESI interface, coupled with an Acquity UPLC system featuring a binary pump and a micro autosampler from Waters (Milford, MA, USA). Interface conditions were set as follows: TEM, 450 °C; CUR, nitrogen, 20 psi; GS1, air, 50 psi; GS2, air, 30 psi; ion spray voltage +5500 V or −4500 V. Multi-mycotoxin quantification in 98 samples of herb and spice extracts was carried out by measuring peak areas in the MRM chromatogram of the quantifier ions and comparing them with the corresponding matrix-matched calibration curves. The seven-point calibration in matrix extracts ranged as follows: 0.01–2 ng injected for FB_1_ and FB_2_; 0.002–0.4 ng injected for AFB_1_, AFG_1_, OTA, and HT-2; 0.0005–0.1 ng injected for AFB_2_ and AFG_2_; 0.004–0.8 ng injected for T-2; 0.001–0.2 ng injected for ZEA; 0.07–14 ng injected for DON.

#### 2.3.3. LC-MS/MS Parameters for the Determination of Multi-Mycotoxins

The MS/MS parameters for the mycotoxins analyzed in this study were provided in Gambacorta et al., 2018 [21]. Each sample extract was analyzed twice, in negative ion mode for DON and ZEA and in positive ion mode for AFB_1_, AFB_2_, AFG_1_, AFG_2_, OTA, FB_1_, FB_2_, T-2, and HT-2.

The optimized MS/MS conditions for multi-mycotoxins are listed in Table 3, and three transitions for confirmation and one transition for quantification were used for all mycotoxins, except for aflatoxins. For AFB_1_, AFB_2_, AFG_1_, and AFG_2_, two transitions for confirmation and one transition for quantification were applied. Separation of the 11 mycotoxins was carried out using an Acquity UPLC BEH phenyl analytical column (2.1 × 150 mm, 1.7 μm particles; Waters), with the column oven set at 40 °C. The mobile phase flow rate was 250 μL/min and the injection volume was 10 μL. A binary linear gradient of acidic MeOH (containing 0.5% acetic acid, 1 mM ammonium acetate) in water (containing 0.5% acetic acid, 1 mM ammonium acetate) was used for separation. For positive ion mode, the gradient was as follows: from 20% to 80% acidic MeOH in 20 min, then held at 80% MeOH for 5 min, followed by a rapid return to 20% MeOH in 0.5 min, and finally equilibrated for 4.5 min before the next run. For negative ion mode, the gradient proceeded from 20% to 80% MeOH in 5 min, maintained at 80% MeOH for 5 min, then returned to 20% MeOH in 0.5 min and equilibrated for 4.5 min before the next run.

### 2.4. Statistical Analysis

The mean, median, and standard deviation of the results were calculated using MS Excel 2013 software (Microsoft Corporation, Redmond, WA, USA), while statistical analyses were performed using GraphPad Instat software, Version 3.00, (Instat, San Diego, CA, USA). Data were subjected to an unpaired *t*-test (one-tailed *p* value) with values considered significantly different if *p* < 0.05.

## 3. Results and Discussion

### 3.1. Method Performance

The method’s performance characteristics were obtained by [4] and are reported in Table 2. Based on the mean recoveries and mean repeatability (RSDr) values, the method performance characteristics were deemed acceptable for the analytes tested. Specifically, the mean recoveries for most of the analyzed mycotoxins ranged from 72% (ZEA) to 105% (FB_2_). However, the mean recoveries for AFB_1_ and AFB_2_ were 60% and 61%, respectively. The repeatability of results was acceptable for all mycotoxins, with values ranging from 1% (FB_1_ and HT-2) to 23% (ZEA). LOD values ranged from 0.07 μg/kg (ZEA) to 70.60 μg/kg (DON) and LOQ values ranged from 0.24 μg/kg (ZEA) to 235.30 μg/kg (DON) (Table 2). In addition, the matrix effect was one of the most important parameters to be investigated during the development of a method in mass spectrometry; this effect can give rise to a signal suppression or enhancement [24]. The matrix effect for the eleven mycotoxins was calculated as the ratio between the slope of the matrix-matched curve and the slope of the calibration curve prepared in the LC mobile phase and multiplied by 100 (%SSE). The results ranged from 178% for FB_2_ to 7.6% for DON.

However, the values of LOD and LOQ of the method were acceptable for all mycotoxins, including AFB_1_ and AFB_2_, thanks to cold precipitation before LC-MS/MS determination and the high performance of the instrument.

### 3.2. Multi-Mycotoxin Occurrence in Herbs and Spices Commercialized in Italy

The results regarding multi-mycotoxin occurrence in Italy in 70 samples of spices, 20 samples of herbs, and 8 samples of mixtures are shown in Table 4. When assessing the regulated mycotoxins (AFB_1_, total aflatoxins, and OTA), the percentage of positive samples varied from 3% to 14% for spices and from 0% to 5% for herbs.

The regulated mycotoxins were not detected in samples of herbs-and-spices mixtures.

For aflatoxins, the percentage of positive spice samples was about double compared to herbs, but for AFB_1_ the percentage of positive samples was higher in herbs and not in spices, whereas for OTA, it was only present in spices with a positive percentage of 14%, while OTA was not present in samples of herbs (Table 4).

The average level of AFB_1_ in positive samples of spices was 6.3 μg/kg, a value just above the European limit of AFB_1_ in some spices (5 μg/kg) laid down in Commission Regulation (EC) No. 165/2010. AFB_2_ was detected in two spice samples at levels below the LOQ (4.3 μg/kg); AFG_2_ was also detected in two spice samples at levels below the LOQ (3.0 μg/kg), whereas AFG_1_ was never detected. For total aflatoxins, the average level (6.3 μg/kg) in the six positive spice samples was below the European limit of 10 μg/kg. In the case of herbs, the situation was less concerning, as only one sample was contaminated with AFB_1_, at a level of 3.6 µg/kg, which is below the European limit. No herb samples were found to be contaminated with AFB_2_, AFG_1_, or AFG_2_.

No samples of herbs-and-spices mixtures were found to be contaminated by AFB_1_, AFB_2_, AFG_1_ and AFG_2_.

OTA was detected in ten samples of spices in the range of 3.4–7.8 μg/kg and the mean level of positive samples (5.0 μg/kg) was below the European limits (15–20 μg/kg) in Commission Regulation No. 1370/2022 [18]. No samples of herbs or herbs-and-spices mixtures were found to be contaminated by OTA (Table 4). While assessing the non-regulated mycotoxins, ZEA was highly present in spices (76% positives), herbs (50% positives), and herbs-and-spices mixtures (37% positives). But the mean ZEA levels in positive spices (4.6 μg/kg), herbs (2.4 μg/kg), and herbs-and-spices mixtures (2.6 μg/kg) were the lowest mean values among the eleven mycotoxins analyzed in this study.

Instead, the FB_1_ mean levels in positive spices (1813.9 μg/kg) and herbs-and-spices mixtures (1071.7 μg/kg) were the highest mean values among the eleven mycotoxins analyzed in this study. For FB_1_, the percentage of positives in spices was 16% and in herbs-and-spices mixtures was 25%. No samples of herbs were found to be contaminated by FB_1_. Herb samples were contaminated by FB_2_, with a percentage of positive samples of 10% and a mean level of 131.4 μg/kg. For FB_2_, higher percentages of positive samples (17% for spices and 50% for herbs-and-spices mixtures) were observed, but at a lower mean level (142.1 μg/kg for spices and 71.0 μg/kg for herbs-and-spices mixtures). A significant mean level was also observed for DON in spices (423.6 μg/kg) and in herbs-and-spices mixtures (335.3 μg/kg). Only two samples of herbs (basil and parsley) were contaminated by DON, but at levels below the LOQ (235.3 μg/kg).

T-2 and HT-2 were only present in spice samples, with a positive percentage of 1% and 3%, respectively, at mean levels ranging between 27.0 μg/kg and 35.9 μg/kg. T-2 and HT-2 were never detected in any of the samples of herbs or herbs-and-spices mixtures.

Only one sample of spice—that is, cloves—showed an AFB_1_ value (9.0 µg/kg) higher than the limits of the European Regulation. No samples of herbs and spices showed concentrations of total AFs and OTA higher than the limits of European Regulation, respectively, equal to 10 µg/kg and 15 µg/kg.

### 3.3. Comparison of Multi-Mycotoxin (Regulated and Non-Regulated) Occurrence in Different Types of Single Spices, Herbs, and Mixtures

Table 5 shows the mean levels of regulated mycotoxins (total aflatoxins and OTA) and the mean levels of the sum of non-regulated mycotoxins in 30 different spices, 1 spice mixture, 10 different herbs, and 1 herb mixture. These results allow us to identify the categories of herbs and spices that are more susceptible to mycotoxin contamination. Individual data of mycotoxins are reported separately in Supplementary Table S1.

Only six samples of spices were contaminated by aflatoxins, but only one sample (cloves) was found to be contaminated with aflatoxins at values higher than the European limit; in particular, cloves were contaminated by AFB_1_ at a concentration of 9 µg/kg. But cloves are not included among the regulated spices by Commission Regulation No. 1881/2006 [16].

Out of three samples of fennel seeds, only one sample was contaminated by AFB_1_, but at levels below the European Regulation limits. One sample of red chili and one sample of garlic contained AFG_2_, with a mean level of 1.5 µg/kg; AFB_2_ was detected in two spice samples (sunflower seeds and paprika) with a mean level of 2.2 µg/kg.

Within the herbs, one sample (rosemary) contained aflatoxins at levels (3.6 µg/kg) below 10 μg/kg; in particular, this sample of rosemary contained only AFB_1_, while AFB_2_, AFG_1_, and AFG_2_ were never detected in herb samples.

While assessing the OTA occurrence, 10 spice samples (Sichuan pepper, licorice, two samples of paprika, two samples of red chili, ginger, and three samples of turmeric) were found to be contaminated, but no sample of spices was contaminated at a level higher than the European limit of 15 μg/kg. The highest OTA mean level was observed in two samples of paprika (7.2 μg/kg) and in two samples of red chili (6.6 μg/kg). Within the herbs, no samples of herbs were found to be contaminated by OTA.

When assessing the non-regulated mycotoxins, 80% of spices and 55% of herbs were found to be contaminated. Among the spices, poppy seeds, hemps seeds, coriander, and cloves samples tested negative for all of the non-regulated mycotoxins analyzed in this study, although cloves contained high levels of aflatoxins (Table 5).

Spices containing high levels (>500 μg/kg) of non-regulated mycotoxins were as follows, in descending order: garlic, red pepper, flax seeds, Sichuan pepper, and sunflower seeds.

Garlic presented the highest level of fumonisins, up to 6691.46 μg/kg of FB_1_ + FB_2_.

High levels (115,231.9 μg/kg) of fumonisins in garlic were previously reported by [4], where the fumonisins concentration was 17-times higher than in garlic samples commercialized in Italy.

The high fumonisins levels found in this and other studies underline the importance of evaluating the origin of fumonisins (in particular FB_1_) contamination of garlic: in fact, two samples of Italian garlic presented high levels of FB_1_ (6316.4 μg/kg and 5203.7 μg/kg). Only one sample of licorice was contaminated by T-2 and HT-2 toxins, with concentrations, respectively, of 27.0 μg/kg and 90.6 μg/kg. Among the herbs, rosemary, thyme, oregano, mint, and herb mixtures were negative for all of the non-regulated mycotoxins analyzed in this study. The highest level of non-regulated mycotoxins was detected in basil (310.7 μg/kg), in which the highest levels of FB_2_ (183.3 μg/kg) and DON (126.6 μg/kg) were found. On the other hand, FB_1_ was never detected in herb samples. Some spices (poppy seeds, hemps seeds, and coriander) and herbs (thyme, oregano, mint, and herb mixture) were totally without mycotoxins (regulated and non-regulated). The results of Table 5 clearly show a higher incidence of positives and higher mycotoxin levels in spices as compared to herbs, but at not alarming levels.

### 3.4. Comparison of Mycotoxins Occurrence in Samples of Herbs and Spices According to the Part of the Plant Used

Different herbs and spices have been classified according to the edible part of the plant used: fruits, seeds, bulbs, roots, berries, buds, bark, and leaves (Table 6). While assessing the regulated mycotoxins, only the buds had a mean AFB_1_ value (9.0 μg/kg) above the regulation limit. AFB_1_ was also detected in seeds and leaves, with a mean level of 3.7 μg/kg and 3.6 μg/kg, respectively.

OTA was only detected in fruits, roots, and berries, and the highest mean level of OTA was observed in fruits (7.0 μg/kg), but below the regulation limit. AFB_1_, total AFs, and OTA were never detected in bulbs and barks. While assessing the non-regulated mycotoxins, the highest mean level of FB_1_ (2438.1 μg/kg) was observed in bulbs (onion and garlic); FB_1_ was also found in garlic and onion by Boonzaaijer et al., 2008 [25], at levels of 5440.0 μg/kg. High levels of FB_1_ and FB_2_ (median concentrations: 401.0 µg/kg and 491.0 µg/kg, respectively) were detected in 77% and 100% of garlic samples by Anjorin et al., 2021 [26]. The highest mean level of FB_2_ (367.8 μg/kg) and the highest mean level of ZEA (9.5 μg/kg) were found in roots (ginseng, cinnamon, and licorice). Moreover, the roots are the only part of plant where T-2 and HT-2 toxins have been detected, with a mean level of 27.0 μg/kg and 60.7 μg/kg, respectively; root contamination with toxigenic fungi and mycotoxins was also demonstrated by Chunyan et al., 2018 [27]. In particular, in a licorice sample, the highest concentrations of AFB_1_ (478.0 μg/kg) and AFs (1330.0 μg/kg) were observed, but T-2 and HT-2 were never detected.

High levels of DON were observed in berries (393.8 μg/kg). Buds and barks were the parts of the plant contaminated with a single mycotoxin: AFB_1_ and ZEA, respectively. The most contaminated parts were bulbs (onion and garlic), berries (different type of pepper), and seeds (different type of seeds, nutmeg, cumin, and coriander) with a mean mycotoxin level of 684.8 μg/kg, 295.2 μg/kg, and 216.7 μg/kg, respectively.

The least contaminated parts were fruits (paprika and red chili) and barks (cinnamon) with a mean mycotoxin level of 4.2 μg/kg and 5.9 μg/kg, respectively. Our findings agree with those of El-Kady et al., 1995; Musaiger et al., 2008; O’Riordan and Wilkinson, 2008; Cho et al., 2008; and Ozbey and Kabak, 2012 [28,29,30,31,32], which showed that cinnamon was not likely to be a good substrate for the growth of mycotoxin-producing fungi and mycotoxin accumulation. The individual levels of mycotoxins are reported separately in Supplementary Table S2.

### 3.5. Comparison of Mycotoxins Occurrence in Samples of Herbs and Spices Commercialized in Italy and in Lebanon

The results of this study were compared with the results obtained by El Darra et al., 2019 [4], which evaluated the presence of the same mycotoxins in herbs and spices commercialized in Lebanon. For the evaluation of the results, results below the LOD were given the value of LOD/2 and results below the LOQ were given the value of LOQ/2 according to the middle-bound approach reported by the EFSA, 2010 [33].

The mean levels of mycotoxins measured in positive samples of herbs and spices (in common) commercialized in Italy and Lebanon are reported in Table 7.

While assessing the regulated mycotoxins, a significant difference was noted for AFB_1_ and total aflatoxins. In Italian spices the mean levels of AFB_1_ and total AFs were about 40- and 15-times lower than Lebanese spices, respectively. Within the herbs, the mean levels of AFB_1_ and total AFs in Lebanese samples were about five- and two-times higher than the Italian herbs, but differences were not significant. The high levels of AFB_1_ and total AFs in Lebanese spices compared to Italian spices suggest that in Italy there is probably a good control of herbs and spices, as regards regulated mycotoxins, thanks to European Regulation.

With regard to OTA, the mean level in Lebanese spices (10 positive samples) was only two-times higher than in Italian spices (8 positive samples) and the difference was not significant. The OTA contamination levels in Italian and Lebanese herbs were quite similar and not alarming, with mean levels of 0.2 µg/kg and 0.5 µg/kg, respectively.

Considering non-regulated mycotoxins in spices, no statistically significant differences were observed for all mycotoxins. In particular, the mean levels of FB_1_, FB_2_, and DON were higher in Lebanese spices than in Italian spices, especially the FB_1_ mean level in Lebanese spices which was 38-times higher, but FB_1_ was detected in 38 Lebanese spices, while in Italy, FB_1_ was detected in only 7 samples. In addition, ZEA was mainly present in Italy, with 42 positive samples compared to Lebanon with only 16 positive samples, although there were no significant differences.

Moreover, in general, the mean mycotoxins levels were similar in Italian and Lebanese herbs, except for FB_1_ which was detected in 11 Lebanese herb samples and was never detected in Italian herbs.

These results indicated that in Italy, the mycotoxins contamination in herbs and spices was less worrying, and there are probably climatic conditions in Italy that limit mycotoxins production in herbs and spices. In fact, Lebanon’s warmer, more humid regions favor mold growth on harvested plant materials, increasing mycotoxin production in herbs and spices [5].

Inadequate drying or poor ventilation in storage facilities can allow molds to proliferate post-harvest, and the differences in cleaning, sorting, and mycotoxin-screening protocols may lead to higher residual levels in one region versus another. While the *p*-values do not show a statistically significant gap, the consistently higher averages and positivity rates in Lebanese samples suggest environmental and post-harvest handling factors play an important role in mycotoxin accumulation [34].

## 4. Conclusions

This study provides useful information about multi-mycotoxin (regulated and non-regulated) occurrence in a consistent number of spices (70), herbs (20), and herbs-and-spices mixtures (8) commercialized in Italy. Spices resulted in more contaminated samples as compared to herb samples. A moderate and not alarming contamination of aflatoxins and OTA was observed in herb and spice samples; only one sample of cloves presented an AFB_1_ level beyond the regulation limit, but cloves are not included in the list of regulated spices. In addition, high concentrations of FB_1_ were observed in different spice samples, such as in some samples of garlic. Thus, to better safeguard public health, it will be essential to regulate FB_1_ and FB_2_ in the herbs and spices most prone to heavy contamination, such as garlic. Surveillance efforts should be broadened to include trichothecenes like T-2 and HT-2, especially in commodities such as licorice where these toxins, though less common, have already appeared at concerning concentrations. At the same time, monitoring programs must evolve beyond single-toxin analyses to evaluate co-contamination because 84% of the analyzed samples of spices were contaminated by 1–4 mycotoxins and 60% of the analyzed samples of herbs were contaminated by 1–4 mycotoxins. Thus, further studies should be conducted to assess the consumption of herbs and spices in Italy to quantify the mycotoxin risk for Italian people deriving from the consumption of this food category. By coupling these regulatory and monitoring enhancements with best-practice drying, storage, and screening procedures at every stage of the supply chain, we can meaningfully reduce the mycotoxin burden and ensure that our herbs and spices remain both flavorful and safe.

## Figures and Tables

**Table 1:** List of 70 spices, 20 herbs, and 8 herbs-and-spices mixtures collected in Italian shops.

Spices (65)		Mixed Spices (5)	Herbs (19)	Mixed Herbs (1)	Mixed Herbs and Spices (8)
Red pepper (1)	Hemp seeds (1)	Mixed pepper (1)	Chives (1)	Mixed herbs (1)	Grilled veal mix (1)
Green pepper (2)	Cumin (2)	Mixed Berberè (1)	Dill (1)		Garam masala (1)
Black pepper (5)	Coriander (1)	Curry (2)	Sage (2)		Garlic, red chili, and parsley (1)
White pepper (3)	Licorice (1)	Mix Creola (1)	Basil (3)		Curry and madras (1)
Sichuan pepper (1)	Fenugreek (1)		Parsley (3)		Mix barbecue (1)
Juniper berries (1)	Paprika (3)		Marjoram (1)		Bruschetta and cheese mix (1)
Sunflower seeds (1)	Red chili (3)		Rosemary (3)		Sapori d’oriente (1)
Pumpkin seeds (1)	Nutmeg (3)		Thyme (1)		Roasted mix (1)
Yellow mustard seeds (1)	Cinnamon (4)		Oregano (3)		
Sesame seeds (2)	Ginger (3)		Mint (1)		
Flax seeds (2)	Turmeric (3)				
Anise seeds (1)	Cloves (2)				
Fennel seeds (3)	Onion (5)				
Cardamom seeds (1)	Garlic (7)				
Poppy seeds (1)					

**Table 2:** Spike levels, recoveries, RSDr, LOD, and LOQ of the 11 mycotoxins calculated by [4].

Mycotoxins	Spike Level (µg/kg)	Recovery (%)	RSDr ^1^ (%)	LOD ^2^ (µg/kg)	LOQ ^3^ (µg/kg)
AFB_1_	15	60	6	1.0	3.4
AFB_2_	4	61	6	1.3	4.3
AFG_1_	15	73	4	0.6	2.0
AFG_2_	4	95	6	0.9	3.0
OTA	10	93	5	0.5	1.5
FB_1_	50	98	1	6.1	20.2
FB_2_	50	105	4	6.6	22.2
T-2	20	98	5	1.7	5.6
HT-2	40	103	1	1.1	3.6
DON	500	98	20	70.6	235.3
ZEA	15	72	23	0.1	0.2

^1^ RSDr = relative standard deviation of repeatability; ^2^ LOD = limit of detection; ^3^ LOQ = limit of quantification.

**Table 3:** MS/MS parameters for mycotoxins detection.

Analyte	Precursor Ion	Q1 (*m/z*)	Q3 (*m/z*)
AFB_1_	[AFB_1_ + H]^+^	313.4	241.2 ^a^ 213.4
AFB_2_	[AFB_2_ + H]^+^	315.3	259.4 287.4 ^a^
AFG_1_	[AFG_1_ + H]^+^	329.2	243.3 ^a^ 311.4
AFG_2_	[AFG_2_ + H]^+^	331.1	245.3 313.3 ^a^
OTA	[OTA + H]^+^	404.2	358.5 257.3 239.2 ^a^
T-2	[T-2 + H]^+^	484.3	215.2 ^a^ 185.3
HT-2	[HT-2 + H]^+^	442.4	215.2 ^a^ 263.5
FB_1_	[FB_1_ + H]^+^	272.4	370.6 334.7 ^a^ 316.6
FB_2_	[FB_2_ + H]^+^	706.4	354.6 336.3 ^a^ 318.5
DON	[DON + CH_3_COO]^−^	355.0	273.3 175.0 ^a^ 131.0
ZEA	[ZEA + H]^−^	317.2	295.2 265.0 59.0 ^a^

^a^ transition used for quantification; Q1: first quadrupole; Q3: third quadrupole.

**Table 4:** Summary of multi-mycotoxin results obtained for spices (70), herbs (20), and mixtures (8) commercialized in Italy. Percentage of positive samples, mean, and median concentrations.

	Regulated Mycotoxins			Non-Regulated Mycotoxins					
Spices (n = 70)	AFB1	Total AFs	OTA	FB1	FB2	T-2	HT-2	ZEA	DON
min (µg/kg)	3.7	3.7	3.4	343.5	27.3	27.0	11.1	0.5	392.8
max (µg/kg)	9.0	9.0	7.8	6316.4	375.0	27.0	60.7	20.4	454.5
mean (µg/kg)	6.3	6.3	5.0	1813.9	142.1	27.0	35.9	4.6	423.7
median (µg/kg)	6.3	6.3	4.2	683.9	104.3	27.0	35.9	3.5	423.7
n. positives	2	6	10	11	12	1	2	53	7
% positive	3	9	14	16	17	1	3	76	10
	**Regulated Mycotoxins**			**Non-Regulated Mycotoxins**					
**Herbs (n = 20)**	**AFB_1_**	**Total AFs**	**OTA**	**FB_1_**	**FB_2_**	**T-2**	**HT-2**	**ZEA**	**DON**
min (µg/kg)	3.6	3.6	na	na	79.4	na	na	0.7	<LOQ
max (µg/kg)	3.6	3.6	na	na	183.4	na	na	5.5	<LOQ
mean (µg/kg)	3.6	3.6	na	na	131.4	na	na	2.4	<LOQ
median (µg/kg)	3.6	3.6	na	na	131.4	na	na	2.5	<LOQ
n. positives	1	1	0	0	2	0	0	10	2
% positive	5	5	0	0	10	0	0	50	10
	**Regulated Mycotoxins**			**Non-Regulated Mycotoxins**					
**Herbs-and-Spices Mixtures (n = 8)**	**AFB_1_**	**Total AFs**	**OTA**	**FB_1_**	**FB_2_**	**T-2**	**HT-2**	**ZEA**	**DON**
min (µg/kg)	na	na	na	1070.1	26.5	na	na	1.6	326.8
max (µg/kg)	na	na	na	1073.4	134.6	na	na	3.2	343.7
mean (µg/kg)	na	na	na	1071.7	71.0	na	na	2.6	335.3
median (µg/kg)	na	na	na	1071.7	61.5	na	na	3	335.3
n. positives	0	0	0	2	4	0	0	3	2
% positive	0	0	0	25	50	0	0	38	25

na: not applicable.

**Table 5:** Occurrence of regulated and non-regulated mycotoxins in herbs and spices and relevant mean levels of positive samples.

Spices	Total AFs		Ochratoxin A		Non-Regulated Mycotoxins ^a^	
	Positive/Total	µg/kg	Positive/Total	µg/kg	Positive/Total	µg/kg
**Cloves**	**1/2**	**9.0**	0/2	nd	0/2	nd
Garlic	1/7	<LOQ	0/7	nd	7/7	2250.5
Red pepper	0/1	nd	0/1	nd	1/1	1632.9
Flax seeds	0/2	nd	0/2	nd	2/2	1409.8
Sichuan pepper	0/1	nd	1/1	3.4	1/1	853.4
Sunflower seeds	1/1	<LOQ	0/1	nd	1/1	603.9
Spices mix	0/3	nd	0/3	nd	2/3	332.9
Turmeric	0/3	nd	3/3	3.8	3/3	139.9
Fennel seeds	1/3	3.7	0/3	nd	1/3	130.8
Cumin	0/2	nd	0/2	nd	1/2	126.7
Licorice	0/1	nd	1/1	4.1	1/1	90.6
Green pepper	0/2	nd	0/2	nd	1/1	65.3
Red chili	1/3	<LOQ	2/3	6.6	2/3	64.5
Pumpkin seeds	0/1	nd	0/1	nd	1/1	46.3
Nutmeg	0/3	nd	0/3	nd	3/3	45.8
Pepper mix	0/2	nd	0/2	nd	2/2	21.6
Sesame seeds	0/2	nd	0/2	nd	2/2	7.6
Cinnamon	0/4	nd	0/4	nd	3/4	5.9
Yellow mustard seeds	0/1	nd	0/1	nd	1/1	5.2
Cardamom seeds	0/1	nd	0/1	nd	1/1	5.1
Onion	0/5	nd	0/5	nd	5/5	4.6
Anise seeds	0/1	nd	0/1	nd	1/1	3.3
Paprika	1/3	<LOQ	2/3	7.2	3/3	2.8
White pepper	0/3	nd	0/3	nd	3/3	2.8
Black pepper	0/5	nd	0/5	nd	4/5	2.3
Juniper berries	0/1	nd	0/1	nd	1/1	2.2
Fenugreek	0/1	nd	0/1	nd	1/1	1.3
Ginger	0/3	nd	1/3	3.5	2/3	1.2
Coriander	0/1	nd	0/1	nd	0/1	nd
Hemp seeds	0/1	nd	0/1	nd	0/1	nd
Poppy seeds	0/1	nd	0/1	nd	0/1	nd
Total	6/70	6.3 ^b^	10/70	4.8 ^b^	56/70	291.1 ^b^
**Herbs**	**Total aflatoxins**		**Ochratoxin A**		**Non-regulated mycotoxins ^a^**	
	**Positive/Total**	**µg/kg**	**Positive/Total**	**µg/kg**	**Positive/Total**	**µg/kg**
Basil	0/3	nd	0/3	nd	3/3	310.7
Parsley	0/3	nd	0/3	nd	3/3	46.0
Sage	0/2	nd	0/2	nd	2/2	40.2
Dill	0/1	nd	0/1	nd	1/1	3.2
Chives	0/1	nd	0/1	nd	1/1	2.3
Marjoram	0/1	nd	0/1	nd	1/1	0.7
Rosemary	1/3	3.6	0/3	nd	0/3	nd
Timo	0/1	nd	0/1	nd	0/1	nd
Oregano	0/3	nd	0/3	nd	0/3	nd
Mint	0/1	nd	0/1	nd	0/1	nd
Herb mixture	0/1	nd	0/1	nd	0/1	nd
Total	1/20	3.6 ^b^	0/20	nd	11/20	67.2 ^b^

nd: not detected. ^a^ Mean of sum of FB_1_, FB_2_, ZEA, DON, T-H, and HT-2. ^b^ Mean of positive samples. Bold text represents the sample exceeding EU regulatory limits and background shading represents mycotoxins with detection rates above 50%.

**Table 6:** Mean levels (μg/kg) of FB_1_, FB_2_, AFB_1_, total aflatoxins, OTA, T-2, HT-2, DON, and ZEA in positive samples of herbs and spices according to the edible part of the plant used.

Mycotoxins	Fruits	Seeds	Bulbs	Roots	Berries	Buds	Bark	Leaves
	µg/kg							
Total AFs	3.7	5.9	nd	nd	nd	**9.0**	nd	3.6
Ochratoxin A	6.9	nd	nd	3.8	3.4	nd	nd	nd
Non-regulated mycotoxins ^a^	2.2	1291.1	2739.1	465.0	1472.6	nd	5.9	260.0

nd: not detected. ^a^ Mean of sum of FB_1_, FB_2_, ZEA, DON, T-H, and HT-2.

**Table 7:** Mean levels (μg/kg) of AFB_1_, total aflatoxins, OTA, FB_1_, FB_2_, T-2, HT-2, DON, and ZEA in positive samples of herbs and spices in common in Italy and Lebanon.

Mycotoxins	Italian Spices		Lebanese Spices		*p* Value
	µg/kg	n of Positives	µg/kg	n of Positives	
**AFB_1_**	0.8	2	34.4	10	**0.0273**
**Total AFs**	2.3	5	37.5	13	**0.0260**
OTA	0.9	8	1.9	10	0.1618
FB_1_	136.1	7	5277.8	38	0.0580
FB_2_	15.6	5	92.6	15	0.0772
T-2	0.8	nd	0.9	1	0.4605
HT-2	0.7	1	0.9	3	0.3757
DON	48.2	6	315.5	8	0.0649
ZEA	3.4	42	7.4	16	0.1516
**Mycotoxins**	**Italian Herbs**		**Lebanese Herbs**		***p* Value**
	**µg/kg**	**n of Positives**	**µg/kg**	**n of Positives**	
AFB_1_	0.6	1	5.5	2	0.1677
Total AFs	2.0	1	6.6	3	0.2421
OTA	0.2	0	0.5	3	na
**FB_1_**	3.0	0	1969.5	11	**0.0139**
FB_2_	15.6	2	4.9	1	0.2561
T-2	0.8	0	0.8	1	0.9085
HT-2	0.5	0	0.6	1	na
DON	42.9	2	10.2	0	na
ZEA	0.7	7	0.1	0	0.2130

na: not applicable. Bold represents statistically different mycotoxins between Italian and Lebanese samples.

## Data Availability

The original contributions presented in this study are included in the article. Further inquiries can be directed to the corresponding author.

## Supplementary Materials

The following supporting information can be downloaded at: https://www.mdpi.com/article/10.3390/foods14111889/s1, Table S1: Occurrence of regulated and not-regulated mycotoxins in spices and herbs and relevant mean levels of positive samples.; Table S2: Mean levels (μg/kg) of FB1, FB2, AFB1, total aflatoxins, OTA, T-2, HT-2, DON, ZEA in positive samples of spices and herbs according to the edible part of the plant used.
