# Supplementary material for: Natural Occurrence of Main Mycotoxins in Herbs and Spices Commercialized in Italy
Source: Foods. 2025 May 26;14(11):1889. doi: 10.3390/foods14111889 (PMC12153999; doi:10.3390/foods14111889)
Supplement: Supplementary file 1 [file foods-14-01889-s001.zip › foods-3634723-supplementary.pdf]

Table S1. Occurrence of regulated and not-regulated mycotoxins in spices and herbs and relevant mean levels of positive samples.

**Spices**	**AFB$_1$**		**Total AFs**		**OTA**		**FB$_1$**		**FB$_2$**		**T-2**		**HT-2**		**DON**		**ZEA**	
	**pos/total**	**µg/kg**	**pos/total**	**µg/kg**	**pos/total**	**µg/kg**	**pos/total**	**µg/kg**	**pos/total**	**µg/kg**	**pos/total**	**µg/kg**	**pos/total**	**µg/kg**	**pos/total**	**µg/kg**	**pos/total**	**µg/kg**
**Cloves**	**1/2**	**9.0**	**1/2**	**9.0**	0/2	nd	0/2	nd	0/2	nd	0/2	nd	0/2	nd	0/2	nd	0/2	nd
Garlic	0/1	nd	1/7	<LOQ	0/7	nd	6/7	2076.7	4/7	170.3	0/7	nd	0/7	nd	1/7	<LOQ	7/7	3.5
Red pepper	0/1	nd	0/1	nd	0/1	nd	1/1	1237.0	0/1	nd	0/1	nd	0/1	nd	1/1	392.8	1/1	3.1
Flax seeds	0/2	nd	0/2	nd	0/2	nd	2/2	1402.3	0/2	nd	0/2	nd	0/2	nd	0/2	nd	2/2	7.5
Sichuan pepper	0/1	nd	0/1	nd	1/1	3.4	1/1	683.8	1/1	168.6	0/1	nd	0/1	nd	0/1	nd	1/1	1.0
Sunflower seeds	0/1	ns	1/1	<LOQ	0/1	nd	1/1	598.9	0/1	nd	0/1	nd	0/1	nd	0/1	nd	1/1	5.0
Spices mix	0/3	nd	0/3	nd	0/3	nd	0/3	nd	2/3	96.9	0/3	nd	0/3	nd	1/3	236.0	1/3	5.6
Turmeric	0/3	nd	0/3	nd	3/3	3.8	0/3	nd	1/3	122.6	0/3	nd	0/3	nd	0/3	nd	1/3	17.3
Fennel seeds	1/3	3.7	1/3	3.7	0/3	nd	0/3	nd	0/3	nd	0/3	nd	0/3	nd	1/3	<LOQ	1/3	4.1
Cumin	0/2	nd	0/2	nd	0/2	nd	0/2	nd	0/2	nd	0/2	nd	0/2	nd	1/2	<LOQ	0/2	nd
Liquorice	0/1	nd	0/1	nd	1/1	4.1	0/1	nd	0/1	nd	1/1	27.0	1/1	60.7	0/1	nd	1/1	2.9
Green pepper	0/2	nd	0/2	nd	0/2	nd	0/2	nd	1/2	65.3	0/2	nd	0/2	nd	0/2	nd	0/2	nd
Red chili	0/1	nd	1/3	<LOQ	2/3	6.6	0/3	nd	0/3	nd	0/3	nd	0/3	nd	1/3	<LOQ	2/3	1.2
Pumpkin seeds	0/1	nd	0/1	nd	0/1	nd	0/1	nd	1/1	38.2	0/1	nd	0/1	nd	0/1	nd	1/1	8.1
Nutmeg	0/3	nd	0/3	nd	0/3	nd	0/3	nd	0/3	nd	0/3	nd	0/3	nd	1/3	<LOQ	3/3	3.6
Pepper mix	0/2	nd	0/2	nd	0/2	nd	0/2	nd	1/2	18.6	0/2	nd	0/2	nd	0/2	nd	2/2	3.0
Sesame seeds	0/2	nd	0/2	nd	0/2	nd	0/2	nd	1/2	3.6	0/2	nd	0/2	nd	0/2	nd	2/2	3.0
Cinnamon	0/4	nd	0/4	nd	0/4	nd	0/4	nd	0/4	nd	0/4	nd	0/4	nd	0/4	nd	3/4	5.9
Yellow mustard seeds	0/1	nd	0/1	nd	0/1	nd	0/1	nd	0/1	nd	0/1	nd	0/1	nd	0/1	nd	1/1	5.2
Cardamom seeds	0/1	nd	0/1	nd	0/1	nd	0/1	nd	0/1	nd	0/1	nd	0/1	nd	0/1	nd	1/1	5.1
Onion	0/5	nd	0/5	nd	0/5	nd	0/5	nd	0/5	nd	0/5	nd	0/5	nd	0/5	nd	5/5	4.6
Anise seeds	0/1	nd	0/1	nd	0/1	nd	0/1	nd	0/1	nd	0/1	nd	0/1	nd	0/1	nd	1/1	3.3
Paprika	1/3	<LOQ	1/3	<LOQ	2/3	7.2	0/3	nd	0/3	nd	0/3	nd	0/3	nd	0/3	nd	3/3	2.8
White pepper	0/3	nd	0/3	nd	0/3	nd	0/3	nd	0/3	nd	0/3	nd	0/3	nd	0/3	nd	3/3	2.8
Black pepper	0/5	nd	0/5	nd	0/5	nd	0/5	nd	0/5	nd	0/5	nd	0/5	nd	0/5	nd	4/5	2.3
Juniper barries	0/1	nd	0/1	nd	0/1	nd	0/1	nd	0/1	nd	0/1	nd	0/1	nd	0/1	nd	1/1	2.2
Fenugreek	0/1	nd	0/1	nd	0/1	nd	0/1	nd	0/1	nd	0/1	nd	0/1	nd	0/1	nd	1/1	1.3
Ginger	0/3	nd	0/3	nd	1/3	3.5	0/3	nd	0/3	nd	0/3	nd	0/3	nd	0/3	nd	2/3	1.2
Coriander	0/1	nd	0/1	nd	0/1	nd	0/1	nd	0/1	nd	0/1	nd	0/1	nd	0/1	nd	0/1	nd
Hemps seeds	0/1	nd	0/1	nd	0/1	nd	0/1	nd	0/1	nd	0/1	nd	0/1	nd	0/1	nd	0/1	nd
Poppy seeds	0/1	nd	0/1	nd	0/1	nd	0/1	nd	0/1	nd	0/1	nd	0/1	nd	0/1	nd	0/1	nd

Herbs	AFB$_1$		Total AFs		OTA		FB$_1$		FB$_2$		T-2		HT-2		DON		ZEA	
	**pos/total**	**µg/kg**	**pos/total**	**µg/kg**	**pos/total**	**µg/kg**	**pos/total**	**µg/kg**	**pos/total**	**µg/kg**	**pos/total**	**µg/kg**	**pos/total**	**µg/kg**	**pos/total**	**µg/kg**	**pos/total**	**µg/kg**
Basil	0/3	nd	0/3	nd	0/3	nd	0/3	nd	1/3	192.7	0/3	nd	0/3	nd	1/3	<LOQ	3/3	0.3
Parsley	0/3	nd	0/3	nd	0/3	nd	0/3	nd	0/3	nd	0/3	nd	0/3	nd	1/3	<LOQ	3/3	3.8
Sage	0/2	nd	0/2	nd	0/2	nd	0/2	nd	1/2	39.2	0/2	nd	0/2	nd	0/2	nd	1/2	1.0
Dill	0/1	nd	0/1	nd	0/1	nd	0/1	nd	0/1	nd	0/1	nd	0/1	nd	0/1	nd	1/1	3.2
Chives	0/1	nd	0/1	nd	0/1	nd	0/1	nd	0/1	nd	0/1	nd	0/1	nd	0/1	nd	1/1	2.3
Marjoram	0/1	nd	0/1	nd	0/1	nd	0/1	nd	0/1	nd	0/1	nd	0/1	nd	0/1	nd	1/1	0.7
Rosemary	1/3	3.6	1/3	3.6	0/3	nd	0/3	nd	0/3	nd	0/3	nd	0/3	nd	0/3	nd	0/3	nd
Timo	0/1	nd	0/1	nd	0/1	nd	0/1	nd	0/1	nd	0/1	nd	0/1	nd	0/1	nd	0/1	nd
Origan	0/3	nd	0/3	nd	0/3	nd	0/3	nd	0/3	nd	0/3	nd	0/3	nd	0/3	nd	0/3	nd
Mint	0/1	nd	0/1	nd	0/1	nd	0/1	nd	0/1	nd	0/1	nd	0/1	nd	0/1	nd	0/1	nd
Herbs mix	0/1	nd	0/1	nd	0/1	nd	0/1	nd	0/1	nd	0/1	nd	0/1	nd	0/1	nd	0/1	nd

nd: not detected

Used bold text for the sample exceeding EU regulatory limits and background shading for mycotoxins with detection rates above 50%.

Table S2. Mean levels (μg/kg) of FB_1, FB_2, AFB_1, total aflatoxins, OTA, T-2, HT-2, DON, ZEA in positive samples of spices and herbs according to the edible part of the plant used.

Mycotoxins	Fruits	Seeds	Bulbs	Roots	Berries	Buds	Bark	Leaves
	μg/kg							
FB_1	nd	1134.5	2438.1	nd	960.5	nd	nd	nd
FB_2	nd	24.7	170.3	367.8	116.9	nd	nd	131.4
AFB_1	nd	3.7	nd	nd	nd	9.0	nd	3.6
Total AFs	3.7	5.9	nd	nd	nd	9.0	nd	3.6
OTA	6.9	nd	nd	3.8	3.4	nd	nd	nd
T-2	nd	nd	nd	27.0	nd	nd	nd	nd
HT-2	<LOQ	nd	nd	60.7	nd	nd	nd	nd
DON	<LOQ	126.7	126.7	nd	392.8	nd	nd	126.6
ZEA	2.2	5.2	4.0	9.5	2.4	nd	5.94	2.0
Mean	4.3	216.7	684.8	93.8	295.2	9.0	5.94	53.5

nd: not detected
